# Paraben concentrations in cat hair samples

**DOI:** 10.2478/jvetres-2026-0036

**Published:** 2026-06-30

**Authors:** Sławomir Gonkowski, Manolis Tzatzarakis, Elena Vakonaki, Thomas Lamprakis, Krystyna Makowska

**Affiliations:** 1 Department of Clinical Physiology, Faculty of Veterinary Medicine, University of Warmia and Mazury in Olsztyn, 10-719 Olsztyn, Poland; 2 Laboratory of Toxicology, School of Medicine, University of Crete, 70013 Heraklion, Greece; 3 Department of Clinical Diagnostics, Faculty of Veterinary Medicine, University of Warmia and Mazury in Olsztyn, 10-719 Olsztyn, Poland

**Keywords:** aggregate exposure, biomonitoring, cats, endocrine disruptors, hyperlocal exposure

## Abstract

**Introduction:**

Parabens, organic substances used as preservatives in various branches of industry, penetrate the environment and living organisms. There is only scant knowledge of cats’ exposure to parabens. Therefore, the aim of the present study was to assess the levels of common industrial parabens in samples of cat hair.

**Material and Methods:**

Concentration levels of four parabens (methylparaben – MeP, ethylparaben – EtP, propylparaben – PrP and butylparaben – BuP) were analysed in cat hair samples (n = 70) using a liquid chromatography–triple quadrupole mass spectrometer. The signalment, body condition score and possibility of outdoor access were recorded for each cat.

**Results:**

All four parabens were found in the cat hair. The concentrations of parabens in pg/mg were as follows: MeP – range 28.7–7,094.7, median 273.5 and mean 620.6 ± 1,193; EtP – range 10.8–6,458.2, median 43.1 and mean 188.2 ± 778.1; PrP – range 6.6–1,793, median 33.2 and mean 107.5 ± 246.9; and BuP – range 8.9–276.5, median 11.5 and mean 27.2 ± 49.5. Statistically significantly higher levels of PrP were noted in strictly indoor cats compared to cats with outdoor access. Statistically significant differences in paraben levels between animals of different sexes, ages and body conditions were not observed.

**Conclusion:**

The obtained results have shown that cats are exposed to parabens to a considerable extent, which suggests that the exposure to these compounds may negatively affect cats’ health.

## Introduction

Parabens are esters of para-hydroxybenzoic acid (from which the name of these compounds derives) which are esterified at the C4 position (8). They differ in the number of carbon atoms and degree of branching in the functional group, and are divided into short-chain (containing one or two carbon atoms) and long-chain (containing three or more carbon atoms) compounds (6). In nature parabens are produced in small quantities by some species of microorganisms and plants and have anti-mould, anti-yest, anti-fungal and anti-bacterial properties (8, 27). Recognition of these properties has led to the synthesis of parabens on a large scale for the needs of various industries since the 1920s (1, 8). Currently, it is estimated that the annual global production of parabens is approximately 8,000 metric tonnes (1). Parabens are commonly used as preservative additives (factors preventing microbial growth and extending product shelf lives) in cosmetics, food, and pharmaceuticals, and the most frequently used compounds of the type are methylparaben (MeP), ethylparaben (EtP), propylparaben (PrP) and butylparaben (BuP) (13).

The large-scale industrial production of parabens has resulted in pollution of the natural environment with them to a significant degree, and their environmental accumulation provides the condition for them to penetrate human and animal organisms through the digestive tract, respiratory system and skin (8). The presence of parabens has been observed in human blood serum, urine, breast milk and seminal plasma (8, 9). Parabens have also been found in various parts of wild aquatic and terrestrial animal bodies (12, 26). One of the most interesting matrices in which the presence of parabens has been confirmed are hair samples (4, 5, 20). The importance of this matrix is related to parabens’ penetration into the hair and long-term accumulation there. It is important to emphasise that the precise mechanism by which parabens bind to the hair structure remains unknown. On the other hand it is known that keratin – the protein that is the primary component of hair – exhibits a high degree of polarity, allowing it to strongly bind chemical compounds containing a phenolic ring and hydroxyl groups (3). Moreover, studies on bisphenols have shown that compounds containing a phenolic ring can bind to hair keratin through hydrogen bonding and hydrophobic interactions (3). Since parabens also contain a phenolic ring in their molecule, it can be assumed that their binding mechanism to keratin is similar to that of bisphenols. The keratin component of hair, lacking in other matrices, makes hair a very good material for testing long-term exposure to these compounds (31). The analysis of other matrices, such as blood serum or urine, in which paraben levels increase quickly after exposure but also decrease soon afterwards, only allows short-term exposure to be assessed (2). Additionally, hair samples may be collected painlessly and stress-free and are easily stored and transported. Therefore, the importance of hair as a matrix in toxicological studies is growing (31). The majority of previous studies concern paraben levels in human hair (3, 4, 19), but this matrix is also increasingly adopted in research on wild and domestic animals (11, 12, 19).

Although it is known that humans and animals are exposed to parabens, the toxicity of these compounds is still under debate (22). Until recently, it was believed that parabens did not affect living organisms. However, newer research has shown that parabens may be harmful (8, 22). A negative impact of parabens on the reproductive, endocrine, nervous and immune systems, and their genotoxic, cytotoxic, carcinogenic and obesogenic effects have been observed (8, 22, 25). These multidirectional harmful effects of parabens have prompted studies on the exposure of humans and animals to these compounds and are an important research field in modern environmental toxicology.

It should be underlined that companion animals are particularly exposed to anthropogenic substances (23). This is because they live directly next to humans and are exposed to the same factors. Pets come into contact with home furnishings, indoor dust and other potential sources of parabens. They are also fed with commercial food and treated using care products that may contain preservatives, including parabens. Therefore, parabens and other endocrine-disrupting chemicals polluting the environment may be challenges for veterinary medicine as factors affecting companion animal health (19, 23). However, until recently, the issues connected with the detriment to animal health of endocrine-disrupting pollutants were often marginalised. The information on cat exposure to parabens is extremely scant and, to the best of the authors’ knowledge, limited to only two studies describing paraben levels in the urine and faeces of cats living in the USA (14, 18). So far, there has been no information published on the exposure of cats to parabens in Europe. Moreover, analysis of long-term exposure of cats to parabens using hair samples has not been studied at all. It should be pointed out that the assessment of cat exposure to parabens is important not only because of the potential impact of these substances on animal health. Cats are considered good sentinels and an indicator species for the presence and level of harmful substances in the home environment (24). This is because cats usually spend their entire lives at home, they are smaller than humans and therefore more exposed to substances contained in indoor dust, and the latency period of health effects resulting from long-term exposure to environmental pollutants is shorter in cats than in humans (24). Therefore, the monitoring of cat exposure to parabens may also indirectly monitor the content of these compounds in the home environment and reveal the associated possible risk for humans.

The aim of the present study was to assess the levels of MeP, EtP, PrP and BuP, parabens commonly used in industry, in hair samples collected from cats from Olsztyn in north-eastern Poland. Additionally, the influence of lifestyle, weight, age and sex of animals on paraben levels in the hair was studied. The obtained results may enhance knowledge not only of the exposure of cats to parabens, but also of the levels of these compounds in the immediate human environment.

### Material and Methods

#### Reagents

The following reagents were used in this study: MeP, EtP, PrP and BuP (all≥99% purity) and ammonium acetate (≥98%) from Sigma-Aldrich (St. Louis, MO, USA), methanol and acetonitrile (LC-MS grade) from Fischer Chemicals (Loughborough, UK), phenobarbital d^5^ (as internal standard – IS) from Supelco (Bellefonte, PA, USA) and ultrapure water produced by Merck’s Direct-Q 3UVwater purification system (Darmstadt, Germany).

#### Sampling

The hair samples were collected from 70 mixed-breed castrated or spayed cats (37 males and 33 females). All animals had private owners, were clinically healthy and lived in Olsztyn, a city in north-eastern Poland. According to Statistics Poland data from December 2024, the human population of Olsztyn is 166,392 and its population density is 1,884 persons/km^2^. The city has rubber (tyre production), wood, furniture and food industries. Sampling was performed in October and November 2023 during veterinary and/or grooming treatments. From all animals, approximately 2 g of hair was cut using metal scissors very close to the skin from the central part of the abdomen. Immediately after collection the hair was put into small containers of dark glass and stored in the dark at room temperature until analysis. The cats included in the study were divided into three age groups based on cat maturation physiology. The divisions were 1) young cats (≤ 2 years, n = 23); 2) adult cats (> 2 years and ≤ 6 years, n = 26); and 3) mature and senior cats (> 6 years, n = 21). The cats were also divided into groups by body condition according to the international cat body condition score system (29). The first group included underweight animals (BCS points 1–4, n = 12), the second was of animals with the correct weight (BCS 5, n = 41) and the third grouped the obese animals (BCS 6–9, n = 17). Additionally, according to information obtained from their owners, the subjects were divided into those which were strictly indoor cats (n = 55) and those with outdoor access (n = 15). The characterisation of animals included in the study is presented in Supplementary Table S1.

#### Paraben extraction

Paraben extraction was performed with the method described previously by Tzatzarakis *et al*. (28) and Gonkowski *et al*. (12). First, external contaminations were removed from the surface of the hair by washing the samples twice in ultrapure water and twice in methanol. Each step of the washing process lasted approximately 1 min. After being washed, the hair was dried at 50°C and cut into pieces a few mm long. Then 100 mg of the hair was mixed with 2 × 2 mL of methanol and 25 ng of IS in a glass tube and extracted in an ultrasonic water bath for 2 × 2 h at a temperature not exceeding 50°C. The obtained extracts were evaporated to dryness under a nitrogen stream at 35°C and reconstituted with 300 μL of methanol. The obtained solution was transferred into 2 mL vials with inserts for liquid chromatography–tandem mass spectrometry analysis, and 3 μL of it was injected into the system.

#### Instrumentation

An 8060 liquid chromatography triple-quadrupole mass-spectrometer system (Shimadzu, Kyoto, Japan) and Shimadzu SP-C18 column (2.1 mm × 150 mm, 2.7 μm) were used for analysis. Analyte separation was performed at a temperature of 30°C with a flow rate of 0.3 mL/min. The mobile phase was 5 mM ammonium acetate as solvent A and acetonitrile as solvent B. The gradient programme was as follows: initially held at 15% solvent B for 1.0 min, raised to 95% solvent B from 1.0 to 16 min and returned to 15% solvent B from 16 to 20 min. Quantitative and qualitative analyses were performed in multiple reaction monitoring mode to track precursor-product ion transitions, using electrospray ionisation in negative mode. The nebulising gas flow was 2.5 L/min and the drying gas and heating gas flows were 10 L/min. The interface, desolvation line and heat-block temperatures were set at 300°C, 200°C and 200°C, respectively. The retention times and monitoring ions mass-to-charge values for particular compounds are presented in Table 1.

**Table 1.** Liquid chromatography and tandem mass spectrometry retention times and monitoring ions mass-to-charge (m/z) values used in the analysis of companion-cat hair samples for paraben content

Paraben	Rt (min)	Precursor m/z	Product ion m/z	Dwell time	Q1 (V)	CE	Q3 (V)
MeP	7.30	151.1	92	20	18	22	18
MeP	7.30	151.1	136.1	20	18	16	25
EtP	8.89	165.1	92	20	13	22	20
EtP	8.89	165.1	137	20	13	17	27
PrP	10.25	179.1	92	20	10	24	18
PrP	10.25	179.1	136.1	20	10	18	12
BuP	11.47	193.3	137.1	20	10	17	12
BuP	11.47	193.3	93	20	11	21	19
Phenobarbital d5 (IS)	7.24	236.1	85	20	26	12	11
Phenobarbital d5 (IS)	7.24	236.1	42	20	13	17	15

1 MeP – methylparaben; EtP – ethylparaben; PrP – propylparaben; BuP – butylparaben; IS – internal standard; Rt – retention time; Precursor – ion selected for fragmentation; Product ion – ion formed after fragmentation of the precursor ion; Dwell time – transition monitoring time; Q1 (V) – quadrupole-1 voltage; CE – collision energy; Q3 (V) – quadrupole-3 voltage

#### Method validation

The efficacy of the method was evaluated. Paraben standard solutions were made at 0, 6.25, 12.5, 25, 100 and 250 ng/mL concentrations, as were spiked sample solutions at 0, 12.5, 25, 50, 100, 250 and 500 pg/mg concentrations. Standard solutions and spiked solutions were analysed. Extrapolation of the calibration curve was applied to determine concentrations that exceeded the upper limit of the calibration range.

The limits of detection (LOD) and quantification (LOQ) were evaluated using the standard error and the slope of the calibration curves in triplicate (n = 3). Four repeats of spiked samples (n = 4) at all tested concentration levels were used for the evaluation of the recovery and the accuracy of the method, as well as the inter-day precision (% relative SD). The analytical and validation parameters of the method are presented in Table 2.

**Table 2.** Validation parameters of the method used for determination of paraben content in companion-cat hair samples

	MeP	EtP	PrP	BuP	N
LOD (pg/mg)	7.5	3.5	1.8	2.9	-
LOQ (pg/mg)	22.8	10.6	5.5	8.9	-
% recovery	79.6	88.5	95.5	99.7	4
% accuracy	104.6	107.7	107.4	106.0	4
Inter day precision (%RSD)	10.9	7.6	8.6	15.4	4
r^2^ standard solutions	0.9959	0.9954	0.9959	0.9945	3
r^2^ spiked samples	0.9950	0.9966	0.9973	0.9981	4

1 MeP – methylparaben; EtP – ethylparaben; PrP – propylparaben; BuP – butylparaben; LOD – limit of detection; LOQ – limit of quantification; RSD – relative SD

#### Statistical analysis

The statistical analysis was performed with GraphPad Prism version 9.2.0 (GraphPad Software, San Diego, CA, USA). Descriptive statistics with evaluation of minimum, the 25^th^ percentile, median, 75^th^ percentile, maximum, arithmetic mean, SD, SEM, geometric mean and geometric SD were used to characterise paraben levels in the hair samples. Because the data showed non-normal distribution, as had been verified earlier using the Shapiro–Wilk test, differences in paraben levels between particular animal groups were evaluated with a non-parametric Mann–Whitney test (comparison of two groups) or Kruskal–Wallis test (comparison of three groups). Values below the LOD and LOQ were included in the statistical analysis as LOD/2 or LOQ/2, respectively.

### Results

#### Method validation

The linearity of the method with the paraben standard solutions was found to be 0.9959 for MeP, 0.9954 for EtP, 0.9959 for PrP and 0.9945 for BuP. For the spiked sample solutions, it was found to be 0.9950 for MeP, 0.9966 for EtP, 0.9973 for PrP and 0.9981 for BuP. The analytical and validation parameters of the method are presented in Table 2.

#### Paraben concentrations in cat hair samples

**Fig. 1.**
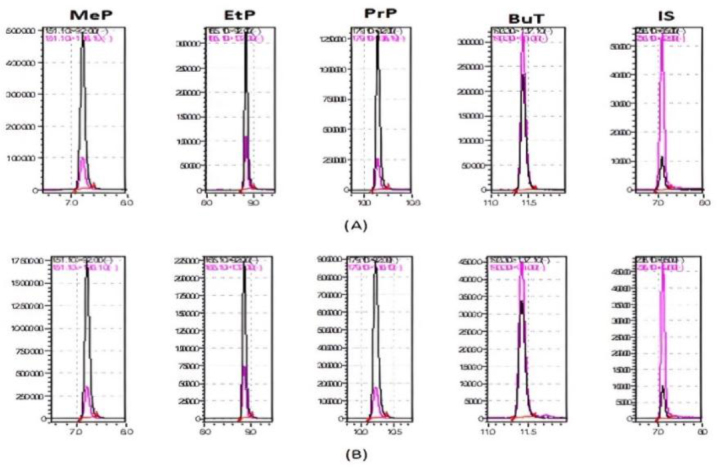
Multiple-reaction monitoring chromatogram of a spiked companion-cat hair sample (at 50 pg/mg) (A) and a cat hair sample positive for methylparaben (MeP) (420.1 pg/mg), ethylparaben (EtP) (60.8 pg/mg), propylparaben (PrP) (71.5 pg/mg) and butylparaben (BuP) (19.3 pg/mg) (B). IS – internal standard

All parabens studied were found in the cat hair samples (Tables 3 and 4, Fig. 1), and three or four in concentrations above their respective LOQs were found in all but four samples; the remaining four samples contained two such compounds (Supplementary Table S2). Very clear differences in paraben concentrations were found between particular animals.

**Table 3.** Concentration levels of parabens (pg/mg) in particular animals included into the study

Animal No.	MeP	EtP	PrP	BuP
cat 01	441.2	89.5	45.9	35.7
cat 02	360.4	25.6	17.7	11.5
cat 03	1276.7	91.0	257.0	64.3
cat 04	437.9	78.0	49.0	15.2
cat 05	179.6	48.4	21.2	9.3
cat 06	7094.7	6458.2	1793.0	27.7
cat 07	609.0	63.6	102.8	19.7
cat 08	677.2	775.2	211.3	49.1
cat 09	377.3	80.0	19.8	11.4
cat 10	208.3	20.1	68.5	12.5
cat 11	406.1	145.6	18.7	<LOQ
cat 12	357.7	77.2	70.9	27.8
cat 13	163.7	12.4	15.0	<LOQ
cat 14	420.1	60.8	71.5	19.3
cat 15	217.5	25.4	27.7	9.6
cat 16	592.2	206.0	110.0	54.0
cat 17	191.0	12.2	18.1	14.4
cat 18	723.5	115.4	71.0	62.7
cat 19	1488.1	128.6	74.0	95.0
cat 20	486.8	10.8	18.7	<LOQ
cat 21	248.0	23.4	24.1	8.9
cat 22	110.4	11.6	11.8	<LOQ
cat 23	86.3	24.6	8.6	<LOQ
cat 24	848.8	23.9	114.0	<LOQ
cat 25	489.2	224.3	96.2	276.4
cat 26	31.8	<LOQ	7.9	<LOD
cat 27	620.1	62.6	80.3	42.3
cat 28	245.0	32.2	166.7	16.7
cat 29	139.8	103.5	12.6	12.7
cat 30	147.0	<LOQ	34.9	<LOQ
cat 31	28.7	<LOQ	8.6	<LOQ
cat 32	228.7	48.2	41.1	31.4
cat 33	200.6	<LOQ	13.0	<LOQ
cat 34	433.1	72.2	84.9	15.7
cat 35	876.4	101.8	73.4	61.2
cat 36	1742.2	421.3	130.9	113.8
cat 37	845.6	175.3	259.9	16.2
cat 38	1399.9	158.5	233.1	12.7
cat 39	1078.5	45.2	196.1	24.4
cat 40	3223.2	292.8	120.6	276.5
cat 41	49.8	<LOQ	28.6	<LOD
cat 42	34.3	<LOQ	11.4	<LOD
cat 43	107.2	17.9	20.7	<LOQ
cat 44	289.6	133.8	35.9	74.9
cat 45	317.1	193.1	55.6	38.4
cat 46	329.8	126.7	71.6	11.4
cat 47	223.1	21.9	15.9	<LOQ
cat 48	115.5	13.1	11.5	<LOD
cat 49	295.6	105.4	42.7	<LOQ
cat 50	94.2	13.9	18.8	<LOD
cat 51	149.8	22.5	26.8	<LOQ
cat 52	183.1	1046.8	1018.2	<LOQ
cat 53	257.3	26.1	30.3	<LOQ
cat 54	105.4	19.8	19.3	10.8
cat 55	49.7	<LOQ	7.1	<LOQ
cat 56	42.9	<LOQ	6.6	<LOD
cat 57	162.7	26.3	8.4	<LOQ
cat 58	104.0	16.7	7.5	<LOQ
cat 59	94.5	25.2	16.5	12.3
cat 60	134.3	32.5	25.7	15.6
cat 61	150.7	34.6	25.8	14.5
cat 62	737.1	385.8	174.7	14.0
cat 63	554.0	42.6	31.5	<LOD
cat 64	574.4	156.0	292.5	10.2
cat 65	758.1	142.1	156.7	31.2
cat 66	93.3	15.8	17.7	9.1
cat 67	105.0	<LOQ	17.1	<LOQ
cat 68	852.5	43.6	116.4	15.3
cat 69	60.5	<LOQ	13.4	<LOQ
cat 70	6684.5	115.5	396.4	100.7

**Table 4.** Paraben levels (pg/mg) and frequency (%) of detection in companion-cat hair samples (n = 70)

	MeP	EtP	PrP	BuP
Minimum (only values > LOQ)	28.7	10.8	6.6	8.9
25^th^ Percentile	129.6	16.5	17.6	<LOQ
Median	273.5	43.1	33.2	11.5
75^th^ Percentile	611.8	118.3	104.6	27.7
Maximum	7,094.7	6,458.2	1,793	276.4
Mean	620.6	188.2	107.5	27.2
SD	1,193	778.1	246.9	49.5
SEM	142.6	93.0	29.5	5.9
Geometric mean	289.4	44.6	43.0	11.6
Geometric SD factor	3.2	4.2	3.4	3.5
% samples >LOD	100	100	100	90
% samples >LOQ	100	85.7	100	64.3

Methylparaben showed the highest concentrations, followed by EtP. Propylparaben concentrations were several-fold lower, and BuP levels were markedly lower across all summary metrics. Methylparaben concentrations ranged from 28.7 pg/mg to 7,094.7 pg/mg, with a mean (±SD) of 620.6 ± 1,193 pg/mg and median of 273.5 pg/mg. Ethylparaben and PrP concentration levels were lower but comparable, while those of BuP were considerably lower, with a mean (±SD) of 27.2 ± 49.5 pg/mg and median of 11.5 pg/mg. Levels above the LOQ were noted in all samples in respect of MeP and PrP, in 85.7% of samples in respect of EtP, and in 64.3% in respect of BuP. Data concerning paraben levels in the analysed cat hair are given in Table 4.

**Fig. 2.**
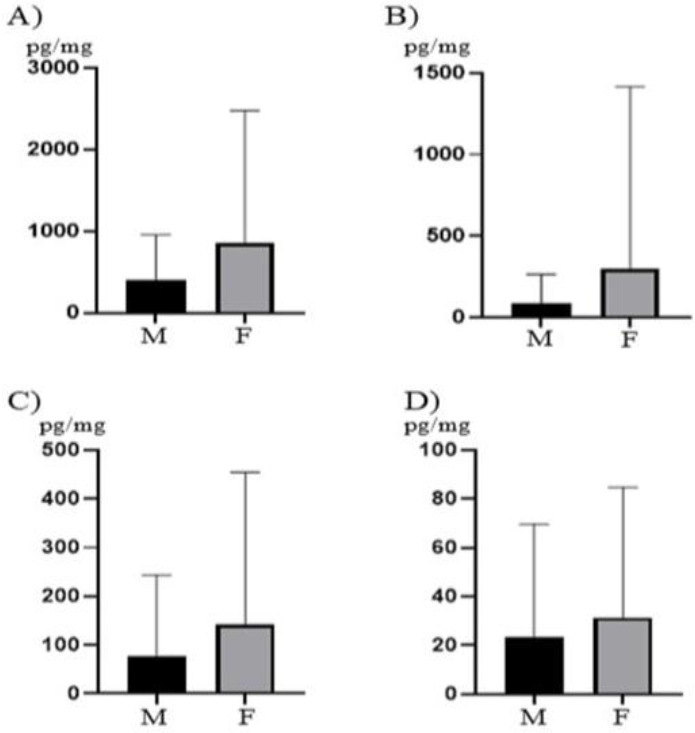
Mean concentration levels (± SD) of (A) methylparaben (MeP); (B) ethylparaben (EtP); (C) propylparaben (PrP) and (D) butylparaben (BuP) in male (M) and female (F) companion-cat hair samples. Statistically significant differences between the sexes were not observed (P-value > 0.05)

Paraben concentrations tended to be higher in females than in males; particular those of MeP and EtP; however, the observed differences were not statistically significant (P-value = 0.5586) (Tables 5 and 6 and Fig. 2A).

**Table 5.** Methylparaben (MeP) and ethylparaben (EtP) levels (pg/mg) in male (n = 37) and female (n = 33) companion-cat hair samples

	Male MeP	Female MeP	Male EtP	Female EtP
Minimum	28.7	34.3	<LOQ	<LOQ
Median	248.0	329.8	26.3	45.2
Maximum	3,223.2	7,094.7	1,046.8	6,458.2
Mean	409.8±547.1	857.0±1,620	89.5±175.3	298.9±1,117

1 LOQ – limit of quantification

**Table 6.** Propylparaben (PrP) and butylparaben (BuP) levels (pg/mg) in male (n = 37) and female (n = 33) companion-cat hair samples

	Male PrP	Female PrP	Male BuP	Female BuP
Minimum	7.5	6.6	<LOD	<LOD
Median	27.7	42.7	11.4	12.7
Maximum	1,018.2	1,793	276.5	276.4
Mean	76.7±167.3	141.9±312.4	23.5±46.2	31.4±53.5

1 LOD – limit of detection

**Fig. 3.**
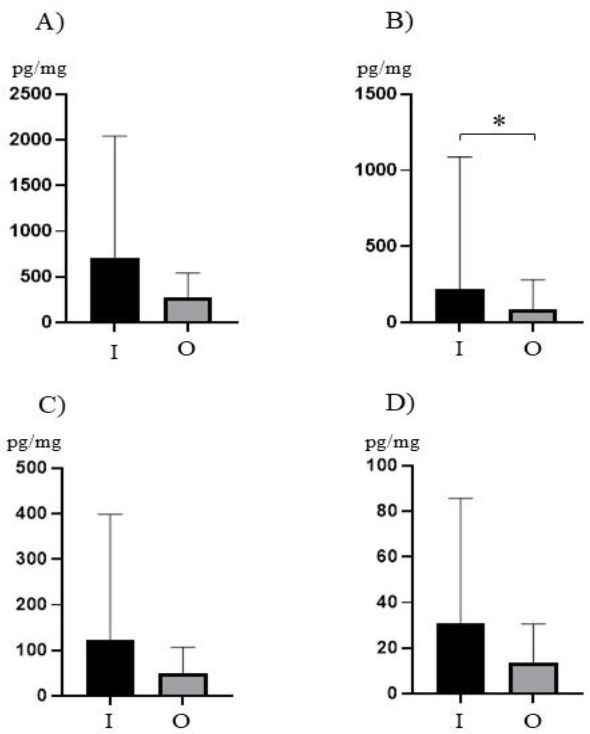
Mean concentration levels (± SD) of (A) methylparaben (MeP); (B) ethylparaben (EtP); (C) propylparaben (PrP); and (D) butylparaben (BuP) in hair samples of indoor (M) and outdoor (O) companion cats. * – statistically significant differences (P-value ≤0.05)

Paraben concentrations tended to be higher in indoor cats compared with those with outdoor access, with statistically significant differences observed only for EtP (p = 0.0175) (Fig. 3) (Tables 7 and 8 and Fig. 3A).

**Table 7.** Methylparaben (MeP) and ethylparaben (EtP) levels (pg/mg) in indoor-restricted and outdoor-accessing companion-cat hair samples

	Indoor MeP	Outdoor MeP	Indoor EtP	Outdoor EtP
Minimum	28.7	31.8	<LOQ	<LOQ
Median	295.6	149.8	60.8	16.7
Maximum	7,094.7	852.5	6,458.2	775.2
Mean	714.4±1326	276.9±265.1	216.9±871.5	83.2±198.2

1 MeP – methylparaben; EtP – ethylparaben; LOQ – limit of quantification

**Table 8.** Propylparaben (PrP) and butylparaben (BuP) levels (pg/mg) in indoor-restricted and outdoor-accessing companion-cat hair samples

	Indoor PrP	Outdoor PrP	Indoor BuP	Outdoor BuP
Minimum	6.6	7.5	<LOD	<LOD
Median	41.1	18.8	12.7	<LOQ
Maximum	1,793	211.3	276.5	54.0
Mean	132.2±275.4	49.7±57.5	30.9±54.7	13.5±17.0

1 PrP – propylparaben; BuP – butylparaben; LOQ – limit of quantification; LOD – limit of detection

**Fig. 4.**
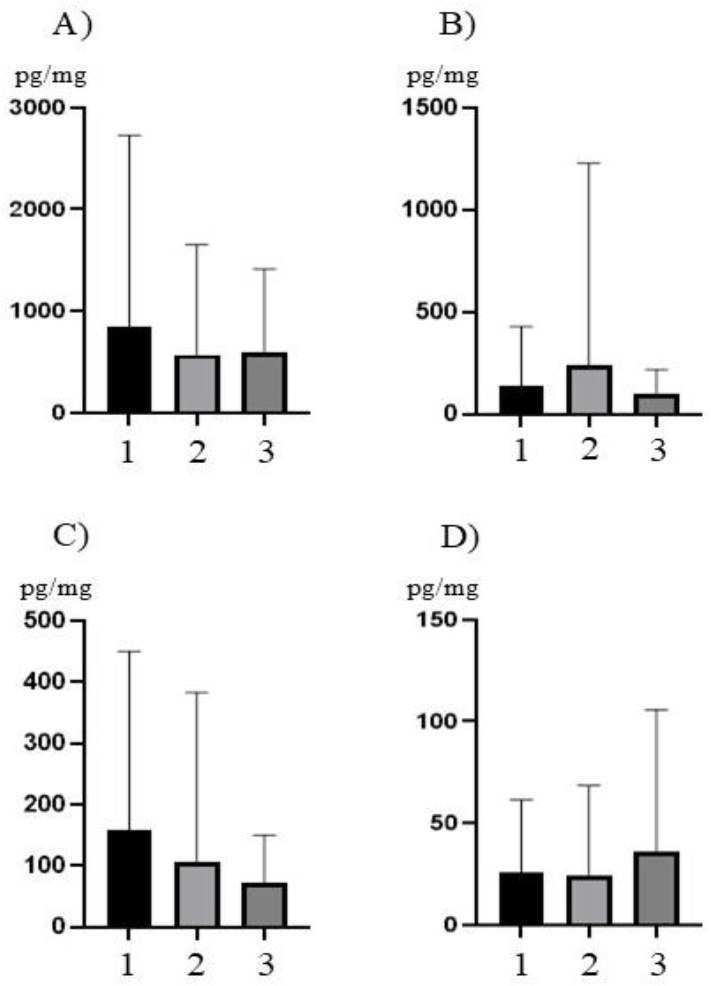
Mean concentration levels (± SD) of (A) methylparaben; (B) ethylparaben; (C) propylparaben; and (D) butylparaben in companion-cat hair samples in (1) underweight animals (body condition score (BCS) 1–4), (2) normal-weight animals (BCS 5) and obese animals (BCS 6–9). Statistically significant differences were not observed (P-value > 0.05)

Paraben concentrations varied across body condition groups, with no statistically significant differences observed for any of the analysed compounds (all P-values > 0.9999) (Tables 9 and 10 and Fig. 4).

**Table 9.** Methylparaben (MeP) and ethylparaben (EtP) levels (pg/mg) in underweight, normal-weight and obese companion-cat hair samples

	Underwt MeP	Normal wt MeP	Obese MeP	Underwt EtP	Normal wt EtP	Obese EtP
Min	31.8	28.7	49.7	<LOQ	<LOQ	<LOQ
Med	218	323.5	716.6	62.8	38.6	34.9
Max	6,684.5	7,094.7	3,223.2	1,046.8	6,458.2	421.3
Mean	848.5±1,875	568±1,086	587.9±825.8	139.3±289.4	236.9±992.3	97.2±121.1

1 MeP – methylparaben; EtP – ethylparaben; LOQ – limit of quantification

**Table 10.** Propylparaben (PrP) and butylparaben (BuP) levels (pg/mg) in underweight, normal-weight and obese companion-cat hair samples

	Underwt PrP	Normal wt PrP	Obese PrP	Underwt BuP	Normal wt BuP	Obese BuP
Min	7.9	6.6	7.1	<LOD	<LOD	<LOD
Med	44.3	29.5	29.5	11.9	11.9	10.8
Max	1,018.2	1,793	292.5	100.7	276.4	276.5
Mean	158.5±291.8	106.6±276.0	71.4±79	26.1±35.3	24.4±44.2	35.6±70.2

1 PrP – propylparaben; BuP – butylparaben; LOD – limit of detection

**Fig 5.**
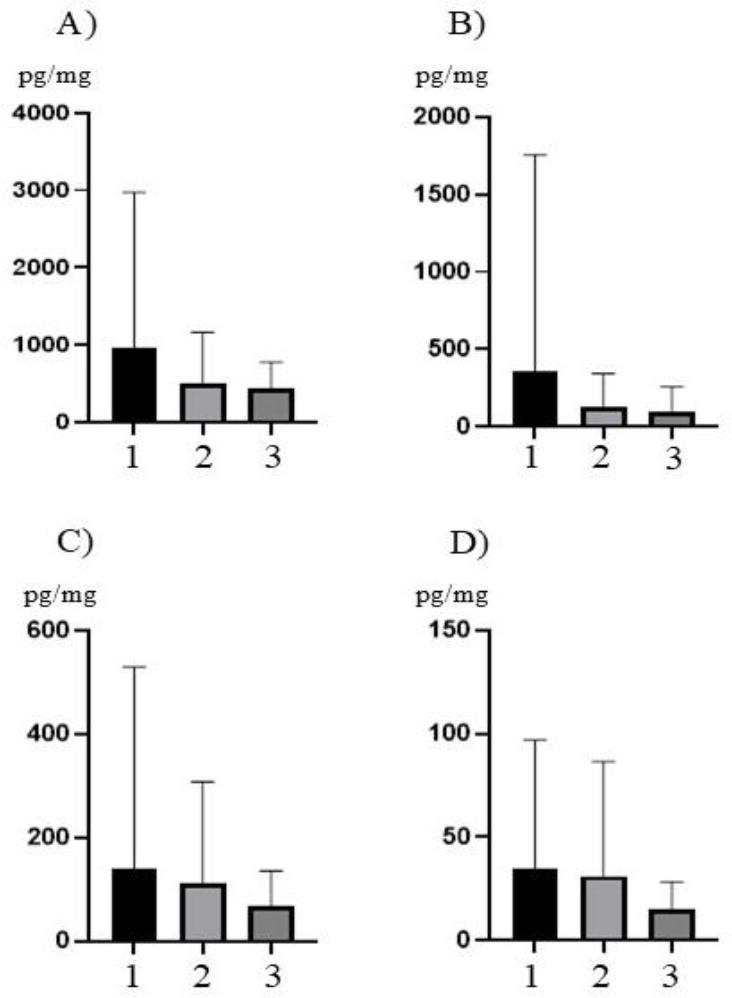
Mean concentration levels (± SD) of (A) methylparaben – MeP; (B) ethylparaben – EtP; (C) propylparaben – PrP; and (D) butylparaben – BuP in companion-cat hair samples from young (1), adult (2) and senior (3) animals. Statistically significant differences were not observed (P-value > 0.05)

Paraben concentrations showed some variation across age groups, with a tendency towards higher levels in younger animals; however, no statistically significant differences were observed (all P-values > 0.9999). (Tables 11 and 12 and Fig. 5).

**Table 11.** Methylparaben (MeP) and ethylparaben (EtP) levels (pg/mg) in young, adult and senior companion-cat hair samples

	Young MeP	Adult MeP	Senior MeP	Young EtP	Adult EtP	Senior EtP
Min	28.7	42.9	49.7	<LOQ	<LOQ	<LOQ
Med	228.7	234.1	377.3	32.5	46.5	62.6
Max	7,094.7	3,223.2	1,399.9	6,458.2	1,046.8	775.2
Mean	966.3±2,005	498.7±664.1	437.4±337.0	359.3±1,399	130.3±213.3	94.8±l62.1

1 MeP – methylparaben; EtP – ethylparaben; LOQ – limit of quantification

**Table 12.** Propylparaben (PrP) and butylparaben (BuP) levels (pg/mg) in young, adult and senior companion-cat hair samples

	Young PrP	Adult PrP	Senior PrP	Young BuP	Adult BuP	Senior BuP
Min	7.9	6.6	7.1	<LOD	<LOQ	<LOQ
Med	25.8	45.8	45.9	12.3	11.4	11.4
Max	1,793	1,018.2	233.1	276.4	276.5	49.1
Mean	140.4±388.9	112.2±195.8	68.2±67.1	34.3±62.5	31.1±55.4	14.9±13.4

1 PrP – propylparaben; BuP – butylparaben; LOD – limit of detection; LOQ – limit of quantification

## Discussion

The results of this study have clearly demonstrated that cats living in Olsztyn are exposed to parabens. Generally, this observation confirms previous research which has described parabens in cats. However, the knowledge on cat exposure to parabens is extremely limited. Only two previous studies carried out in the USA concerned feline paraben levels, and they used urine and faeces samples (14, 18). Unfortunately, the comparison of present results with previous observations in cats is practically impossible. Firstly, because previous studies were performed in regions of the world distant from Poland. It is relatively well known that paraben levels in the environment and living organisms are particular to the region where a study was conducted, because they correlate with the degree of urbanisation and industrialisation and the lifestyle, diet or habits of its residents (8, 22). The second important reason why the current results cannot be compared with the earlier ones is the extraction of the previous analytes from matrices which were not cat hair. Both urine and faeces are matrices which reflect short-term exposure to parabens and other environmental pollutants. Previous studies have shown that paraben levels in these matrices quickly increase after exposure and then equally quickly decrease (2). In contrast to urine or faeces, hair samples reflect long-term exposure. Pollutants (including parabens) accumulate in the hair and their presence can be detected for a long time (31). This explains why a previous study has reported visible differences between the levels of parabens in urine or blood serum and the levels in hair when all three samples were collected from the same individuals (4). Previous studies have shown that in cat urine, the highest concentrations were noted for MeP and were the upper part of concentrations ranging from 150 pg/mL to 65,300 pg/mL (mean 4,200 ± 11,600 pg/mL, median 2,000 pg/mL) (14). Concentrations of EtP, PrP and BuP in cat urine did not exceed 1,000 pg/mL, and their means and medians did not exceed 200 pg/mL (14, 18). In cat faeces means for MeP, EtP, PrP and BuP were 51 ± 60.2 pg/mg (median 37 pg/mg), 6,37 ± 5.02 pg/mg (median 4.21 pg/mg), 10.7 ± 17.0pg/mg (median 4.27 pg/mg) and 0.43 ± 0.26 pg/mg (median <LOD), respectively (18). Direct numerical comparison of paraben concentrations between hair and urine is problematic, as concentrations are expressed per unit mass (pg/mg) in hair and per unit volume (pg/mL) in urine. In turn, it does appear that the paraben levels in hair are higher than those noted in the faeces, despite fundamental differences between these matrices.

The differences referred to may be attributed to several factors. Geographical and lifestyle-related variability can influence paraben exposure, which in consequence is proportional to levels of parabens in the particular environment, in the food given to cats in different regions by owners making different selections, in individual toys played with by animals and in the assortment of care products used on the cat, and is also partially determined by other local factors. However, the differences may be explained by the manner of accumulation of parabens in the hair, as noted above. Substances penetrate into the hair two ways, as opposed to the single way substances reach urine and faeces: internally, through blood vessels and hair bulbs; and externally, directly from the environment (31). The existence of two routes may cause the typical levels of parabens in hair to be higher than those in serum, faeces or urine. However, some similarities between the present results and previous observations are also visible. Namely, both in previous studies and the present observations, higher concentrations in cat samples have been found of MeP, EtP and PrP than of BuP (14, 18). This is because these three parabens are the most widely used in industry, and therefore they are the parabens which pollute the environment most often and to the highest extent.

Given the temporal and mechanistic dissimilarities between paraben accumulation in urine, serum and faeces and their accumulation in hair, it is more appropriate to compare the present results with previous studies on paraben levels in hair, even across species. Analysing previous data concerning parabens in human hair, interregional differences are clearly visible. For example, the median concentration of MeP amounted to 28.9 pg/mg in Belgium (4), 822.1 pg/mg in Spain (20) and even 1,437,100 pg/g in Greece (15). The concentration levels of other parabens in human hair, although much lower than MeP concentrations, also showed large interregional differences (4, 20, 30). As mentioned above, they may be due to various local factors, such as the nature of the regional industries, use of plastics in a particular country, lifestyle, type of agriculture, *etc*. Extreme differences in paraben levels between particular individuals from the same region are also noteworthy (4, 5, 15). They prove the existence of local factors influencing exposure to parabens. Therefore, it is most interesting to compare studies performed in the same area. Previous studies have described the levels of parabens in hair collected from humans and dogs living in the same city as the cats included into this study (Olsztyn) (19, 30). Paraben levels in the cat hair sampled for the present research were lower than those noted previously in local human subjects (30). This seems logical, because parabens are anthropogenic substances and humans may be more exposed to these substances than cats. Higher human exposure may be related to occupational exposure, use of cosmetics or wearing of clothing containing parabens. However, the levels of parabens noted in cats in this study were higher than levels noted in dogs in the same area (19). Interestingly, previous studies have generally found lower levels of parabens in urine and faeces from cats than in these samples from dogs, and this finding has been explained by differences in the metabolism of parabens between these two species (14, 18). Higher paraben levels in cat hair than in dog hair from the same area may suggest that cats are better sentinel and indicator species than dogs regarding the concentration of pollutants (including parabens) in the home environment, which is consistent with previous suggestions (24). Paraben levels in cat hair were also higher than those noted in farm and wild animals (10, 12). This is most likely because cats, as animals living next to humans, come into contact with more sources of parabens.

During the present study statistically significant higher levels of EtP were observed in strictly indoor cats than in cats with outdoor access. Other paraben levels were also higher in indoor cats, but the differences were not statistically significant. The obtained results suggest that home environment is polluted with parabens to a large extent. Previous studies established the main sources of the exposure of pets to parabens to be food (14) and indoor dust (16). Indoor cats are also exposed to parabens and other pollutants by contact with various home appliances, toys, care products, *etc*. (23). The present results are in agreement with previous observations showing higher levels of bisphenol A – another endocrine-disrupting chemical – in indoor cats than in cats with outdoor access (17). In this study the comparison of paraben levels in animals of both sexes, different ages, and better and worse body conditions has not shown any statistically significant differences.

It should be pointed out that knowledge of variations in paraben accumulation across population subgroups is fragmentary and inconclusive. A study on humans concerning paraben levels in each sex has described higher concentrations of these compounds in women than in men, and has explained them by the more frequent use of cosmetics and care products by women (20). Other research on humans has not detected sex differences in paraben levels (30). Data on sex differences in paraben concentrations in animals are also ambiguous. Studies on wild animals and some observations on dogs and cats have no detected such differences (12, 14). Other studies on dogs have reported higher levels of parabens in males, which were posited to result from probable differences in paraben metabolism and from known differences in metabolic rate and hormonal activity between male and female dogs (19). However, the present investigation does not confirm these assumptions in cats, which is in agreement with a previous study analysing urine samples (14). Previous results potentially showing correlations between paraben levels and animal age are also ambiguous. Makowska *et al*. (19) found statistically significantly higher levels of PrP in younger dogs compared to older ones, but other studies on dogs, cats and cows did not describe significant differences in paraben levels between animals of different ages (11, 14). On the other hand, Karthikraj *et al*. (14) showed higher levels of parabens in the urine of younger dogs and cats compared to older ones, albeit none of them statistically significantly different, which has also been found in the present study. Differences in paraben levels in animals of different ages may result from age-dependent changes in metabolism rate and hormonal activity (19). During this study, statistically significant differences in paraben levels between animals of different weights have not been observed. This confounded expectations, because parabens are relatively well known obesogens in humans and previous studies have described correlations between paraben levels and the risk of obesity (25). Parabens may increase the risk of obesity by affecting various processes, such as gluconeogenesis, glycogenolysis and adipogenesis (25). However, the present results may suggest that obesogenic activity of parabens in cats is of less importance than it is in humans.

So far, the negative impact of parabens on the health of cats has not been described. Therefore, it is difficult to determine whether the levels of parabens observed in cat hair in this study may have a harmful effect on animals, especially since the toxicity of parabens, even in humans, is not fully explained and still under discussion (16, 22). On the other hand, multidirectional toxic impacts of parabens are being described more and more often (8). Moreover, it is known that even small doses of parabens may be harmful (7). It should also be remembered that companion animals are usually simultaneously exposed to many harmful endocrine-disrupting substances polluting the environment, which often act synergistically (23). Although it was demonstrated in a non-feline context, research has demonstrated that even very low doses of individual substances can have a negative impact on the body’s health (21). For these reasons, it cannot be ruled out that the levels of parabens observed in this work may have a negative impact on the health of cats. However, a detailed explanation of any actual health detriment requires further comprehensive toxicological, metabolic and clinical studies.

## Conclusion

This study has indicated the presence of various parabens in cat hair, which are one of the best matrices for evaluation of long-term exposure to environmental pollutants. Because of differences between hair and other matrices, one being those other matrices’ reflection only of short-term exposure, a comparison of the present results with previous observations of paraben levels in cat urine and faeces samples is practically impossible. The relatively high paraben levels noted during the study suggest that cats are exposed to these substances to a large extent. Significant differences in the concentration of parabens were found between individual animals, which indicates the existence of hyperlocal factors influencing the degree of exposure to these substances. Higher levels of parabens have been observed in strictly indoor cats than in cats with outdoor access, although statistically significant differences between these animal groups applied only to EtP. These observations suggest a high level of contamination of the home environment with parabens. No correlations have been found between the levels of parabens and the sex, age or body condition of the animals. The relatively high paraben levels noted in cat hair may suggest that these substances can negatively affect the health of cats. However, further comprehensive toxicological, environmental and clinical studies are needed to clarify all aspects related to the impact of parabens on the cat.

## Supplementary Material

Supplementary Material Details

Supplementary Material Details

Supplementary Material Details

Supplementary Material Details
