# Supplementary material for: Paraben concentrations in cat hair samples
Source: J Vet Res. 2026 Jun 30;70(2):341–51. doi: 10.2478/jvetres-2026-0036 (PMC13334297; doi:10.2478/jvetres-2026-0036)
Supplement: Supplementary file 1 — Supplementary Material Details [file jvetres-2026-0036_sm.pdf]

Supplementary Table S1. Characterisation of companion cats included in the hair paraben content study

Cat No.	Sex	Age in years	Outdoor (O) or indoor (I) cat	Body condition score
1	male	7	I	3
2	male	1	O	5
3	female	5	I	5
4	male	8	I	5
5	male	12	I	4
6	female	1	I	5
7	male	13	I	5
8	female	8	O	5
9	male	7	I	6
10	male	4	O	6
11	male	1	I	5
12	female	2.5	O	3
13	female	14	I	5
14	male	5	I	5
15	male	5	I	5
16	male	6	O	7
17	male	1.5	I	3
18	female	1	I	5
19	female	1	I	4
20	male	5.5	O	6
21	male	8	I	7
22	male	9	I	6
23	female	10	I	5
24	female	7	I	5
25	female	1.5	I	5
26	male	1	O	4
27	male	7	I	5
28	male	4	I	3
29	female	6	I	7
30	male	6	I	7
31	male	1	I	5
32	female	1.5	I	5
33	male	5	I	5
34	male	10	I	5
35	male	4	I	5
36	female	5	I	7

37	female	4	I	5
38	male	15	I	5
39	female	1.5	I	5
40	male	5	I	6
41	female	1	O	3
42	female	2	O	4
43	female	1.5	I	5
44	male	6	I	5
45	male	4	I	5
46	female	7	I	5
47	male	5	I	5
48	male	5	I	7
49	female	10	I	3
50	female	2.5	O	5
51	female	2.5	O	5
52	male	6	I	4
53	male	2	I	5
54	male	2	I	5
55	female	11	I	7
56	female	5	I	5
57	male	14	I	7
58	male	7	O	5
59	male	2	I	5
60	female	2	I	5
61	female	1	I	5
62	female	5	I	5
63	female	2	I	5
64	female	5	I	7
65	male	7	I	6
66	female	5	O	5
67	female	5	I	6
68	female	7	O	6
69	male	4	O	5
70	female	2	I	4
