# Supplementary material for: Paraben concentrations in cat hair samples
Source: J Vet Res. 2026 Jun 30;70(2):341–51. doi: 10.2478/jvetres-2026-0036 (PMC13334297; doi:10.2478/jvetres-2026-0036)
Supplement: Supplementary file 2 — Supplementary Material Details [file jvetres-2026-0036_sm1.pdf]

Supplementary Table S2. Concentration levels of parabens (pg/mg) in hair samples of each enrolled companion cat

Cat No.	MeP	EtP	PrP	BuP
01	441.2	89.5	45.9	35.7
02	360.4	25.6	17.7	11.5
03	1276.7	91.0	257.0	64.3
04	437.9	78.0	49.0	15.2
05	179.6	48.4	21.2	9.3
06	7,094.7	6,458.2	1,793.0	27.7
07	609.0	63.6	102.8	19.7
08	677.2	775.2	211.3	49.1
09	377.3	80.0	19.8	11.4
10	208.3	20.1	68.5	12.5
11	406.1	145.6	18.7	<LOQ
12	357.7	77.2	70.9	27.8
13	163.7	12.4	15.0	<LOQ
14	420.1	60.8	71.5	19.3
15	217.5	25.4	27.7	9.6
16	592.2	206.0	110.0	54.0
17	191.0	12.2	18.1	14.4
18	723.5	115.4	71.0	62.7
19	1,488.1	128.6	74.0	95.0
20	486.8	10.8	18.7	<LOQ
21	248.0	23.4	24.1	8.9
22	110.4	11.6	11.8	<LOQ
23	86.3	24.6	8.6	<LOQ
24	848.8	23.9	114.0	<LOQ
25	489.2	224.3	96.2	276.4
26	31.8	<LOQ	7.9	<LOD
27	620.1	62.6	80.3	42.3
28	245.0	32.2	166.7	16.7
29	139.8	103.5	12.6	12.7
30	147.0	<LOQ	34.9	<LOQ
31	28.7	<LOQ	8.6	<LOQ
32	228.7	48.2	41.1	31.4
33	200.6	<LOQ	13.0	<LOQ
34	433.1	72.2	84.9	15.7
35	876.4	101.8	73.4	61.2
36	1742.2	421.3	130.9	113.8
37	845.6	175.3	259.9	16.2
38	1,399.9	158.5	233.1	12.7
39	1,078.5	45.2	196.1	24.4
40	3223.2	292.8	120.6	276.5
41	49.8	<LOQ	28.6	<LOD
42	34.3	<LOQ	11.4	<LOD
43	107.2	17.9	20.7	<LOQ
44	289.6	133.8	35.9	74.9
45	317.1	193.1	55.6	38.4
46	329.8	126.7	71.6	11.4
47	223.1	21.9	15.9	<LOQ

48	115.5	13.1	11.5	<LOD
49	295.6	105.4	42.7	<LOQ
50	94.2	13.9	18.8	<LOD
51	149.8	22.5	26.8	<LOQ
52	183.1	1046.8	1,018.2	<LOQ
53	257.3	26.1	30.3	<LOQ
54	105.4	19.8	19.3	10.8
55	49.7	<LOQ	7.1	<LOQ
56	42.9	<LOQ	6.6	<LOD
57	162.7	26.3	8.4	<LOQ
58	104.0	16.7	7.5	<LOQ
59	94.5	25.2	16.5	12.3
60	134.3	32.5	25.7	15.6
61	150.7	34.6	25.8	14.5
62	737.1	385.8	174.7	14.0
63	554.0	42.6	31.5	<LOD
64	574.4	156.0	292.5	10.2
65	758.1	142.1	156.7	31.2
66	93.3	15.8	17.7	9.1
67	105.0	<LOQ	17.1	<LOQ
68	852.5	43.6	116.4	15.3
69	60.5	<LOQ	13.4	<LOQ
70	6684.5	115.5	396.4	100.7

MeP – methylparaben; EtP – ethylparaben; PrP – propylparaben; BuP – butylparaben; LOQ – limit of quantification; LOD – limit of detection
