# Supplementary material for: Paraben concentrations in cat hair samples
Source: J Vet Res. 2026 Jun 30;70(2):341–51. doi: 10.2478/jvetres-2026-0036 (PMC13334297; doi:10.2478/jvetres-2026-0036)
Supplement: Supplementary file 3 — Supplementary Material Details [file jvetres-2026-0036_sm2.docx]

**Supplementary Table S2.** Concentration levels of parabens (pg/mg) in hair samples of each enrolled companion cat

| Cat No. | MeP | EtP | PrP | BuP |
| --- | --- | --- | --- | --- |
| 01 | 441.2 | 89.5 | 45.9 | 35.7 |
| 02 | 360.4 | 25.6 | 17.7 | 11.5 |
| 03 | 1276.7 | 91.0 | 257.0 | 64.3 |
| 04 | 437.9 | 78.0 | 49.0 | 15.2 |
| 05 | 179.6 | 48.4 | 21.2 | 9.3 |
| 06 | 7,094.7 | 6,458.2 | 1,793.0 | 27.7 |
| 07 | 609.0 | 63.6 | 102.8 | 19.7 |
| 08 | 677.2 | 775.2 | 211.3 | 49.1 |
| 09 | 377.3 | 80.0 | 19.8 | 11.4 |
| 10 | 208.3 | 20.1 | 68.5 | 12.5 |
| 11 | 406.1 | 145.6 | 18.7 | <LOQ |
| 12 | 357.7 | 77.2 | 70.9 | 27.8 |
| 13 | 163.7 | 12.4 | 15.0 | <LOQ |
| 14 | 420.1 | 60.8 | 71.5 | 19.3 |
| 15 | 217.5 | 25.4 | 27.7 | 9.6 |
| 16 | 592.2 | 206.0 | 110.0 | 54.0 |
| 17 | 191.0 | 12.2 | 18.1 | 14.4 |
| 18 | 723.5 | 115.4 | 71.0 | 62.7 |
| 19 | 1,488.1 | 128.6 | 74.0 | 95.0 |
| 20 | 486.8 | 10.8 | 18.7 | <LOQ |
| 21 | 248.0 | 23.4 | 24.1 | 8.9 |
| 22 | 110.4 | 11.6 | 11.8 | <LOQ |
| 23 | 86.3 | 24.6 | 8.6 | <LOQ |
| 24 | 848.8 | 23.9 | 114.0 | <LOQ |
| 25 | 489.2 | 224.3 | 96.2 | 276.4 |
| 26 | 31.8 | <LOQ | 7.9 | <LOD |
| 27 | 620.1 | 62.6 | 80.3 | 42.3 |
| 28 | 245.0 | 32.2 | 166.7 | 16.7 |
| 29 | 139.8 | 103.5 | 12.6 | 12.7 |
| 30 | 147.0 | <LOQ | 34.9 | <LOQ |
| 31 | 28.7 | <LOQ | 8.6 | <LOQ |
| 32 | 228.7 | 48.2 | 41.1 | 31.4 |
| 33 | 200.6 | <LOQ | 13.0 | <LOQ |
| 34 | 433.1 | 72.2 | 84.9 | 15.7 |
| 35 | 876.4 | 101.8 | 73.4 | 61.2 |
| 36 | 1742.2 | 421.3 | 130.9 | 113.8 |
| 37 | 845.6 | 175.3 | 259.9 | 16.2 |
| 38 | 1,399.9 | 158.5 | 233.1 | 12.7 |
| 39 | 1,078.5 | 45.2 | 196.1 | 24.4 |
| 40 | 3223.2 | 292.8 | 120.6 | 276.5 |
| 41 | 49.8 | <LOQ | 28.6 | <LOD |
| 42 | 34.3 | <LOQ | 11.4 | <LOD |
| 43 | 107.2 | 17.9 | 20.7 | <LOQ |
| 44 | 289.6 | 133.8 | 35.9 | 74.9 |
| 45 | 317.1 | 193.1 | 55.6 | 38.4 |
| 46 | 329.8 | 126.7 | 71.6 | 11.4 |
| 47 | 223.1 | 21.9 | 15.9 | <LOQ |
| 48 | 115.5 | 13.1 | 11.5 | <LOD |
| 49 | 295.6 | 105.4 | 42.7 | <LOQ |
| 50 | 94.2 | 13.9 | 18.8 | <LOD |
| 51 | 149.8 | 22.5 | 26.8 | <LOQ |
| 52 | 183.1 | 1046.8 | 1,018.2 | <LOQ |
| 53 | 257.3 | 26.1 | 30.3 | <LOQ |
| 54 | 105.4 | 19.8 | 19.3 | 10.8 |
| 55 | 49.7 | <LOQ | 7.1 | <LOQ |
| 56 | 42.9 | <LOQ | 6.6 | <LOD |
| 57 | 162.7 | 26.3 | 8.4 | <LOQ |
| 58 | 104.0 | 16.7 | 7.5 | <LOQ |
| 59 | 94.5 | 25.2 | 16.5 | 12.3 |
| 60 | 134.3 | 32.5 | 25.7 | 15.6 |
| 61 | 150.7 | 34.6 | 25.8 | 14.5 |
| 62 | 737.1 | 385.8 | 174.7 | 14.0 |
| 63 | 554.0 | 42.6 | 31.5 | <LOD |
| 64 | 574.4 | 156.0 | 292.5 | 10.2 |
| 65 | 758.1 | 142.1 | 156.7 | 31.2 |
| 66 | 93.3 | 15.8 | 17.7 | 9.1 |
| 67 | 105.0 | <LOQ | 17.1 | <LOQ |
| 68 | 852.5 | 43.6 | 116.4 | 15.3 |
| 69 | 60.5 | <LOQ | 13.4 | <LOQ |
| 70 | 6684.5 | 115.5 | 396.4 | 100.7 |

MeP – methylparaben; EtP – ethylparaben; PrP – propylparaben; BuP – butylparaben; LOQ – limit of quantification; LOD – limit of detection
